# The Seminal fluid proteome of the polyandrous Red junglefowl offers insights into the molecular basis of fertility, reproductive ageing and domestication

**DOI:** 10.1038/srep35864

**Published:** 2016-11-02

**Authors:** Kirill Borziak, Aitor Álvarez-Fernández, Timothy L. Karr, Tommaso Pizzari, Steve Dorus

**Affiliations:** 1 Center for Reproductive Evolution, Department of Biology, Syracuse University, US; 2 Edward Grey Institute, Department of Zoology, University of Oxford, UK; 3 Drosophila Genetic Resource Center, Kyoto Institute of Technology, Saga Ippongi-cho, Ukyo-ku, Kyoto 616-8354, Japan

## Abstract

Seminal fluid proteins (SFPs) are emerging as fundamental contributors to sexual selection given their role in post-mating reproductive events, particularly in polyandrous species where the ejaculates of different males compete for fertilisation. SFP identification however remains taxonomically limited and little is known about avian SFPs, despite extensive work on sexual selection in birds. We characterize the SF proteome of the polyandrous Red junglefowl, *Gallus gallus*, the wild species that gave rise to the domestic chicken. We identify 1,141 SFPs, including proteins involved in immunity and antimicrobial defences, sperm maturation, and fertilisation, revealing a functionally complex SF proteome. This includes a predominant contribution of blood plasma proteins that is conserved with human SF. By comparing the proteome of young and old males with fast or slow sperm velocity in a balanced design, we identify proteins associated with ageing and sperm velocity, and show that old males that retain high sperm velocity have distinct proteome characteristics. SFP comparisons with domestic chickens revealed both qualitative and quantitative differences likely associated with domestication and artificial selection. Collectively, these results shed light onto the functional complexity of avian SF, and provide a platform for molecular studies of fertility, reproductive ageing, and domestication.

Seminal fluid (SF), the physiological, non-cellular component of an ejaculate, contains a diversity of molecules including peptides and proteins^1^. A growing body of empirical work has demonstrated the involvement of several such molecules in different stages of reproduction, including sperm fertilising efficiency and longevity, antimicrobial defences, female sperm utilisation, the coordination of ovulation and oviposition in females, maternal allocation, and female remating propensity^2^. Given their modulation of postcopulatory reproductive events, much research has focused on the role of seminal fluid proteins (SFPs) in polyandrous species, where females obtain ejaculates from multiple partners, prolonging sexual selection on males after copulation via sperm competition and cryptic female choice^1^.

Increasing evidence indicates that SFPs can play a key role in postcopulatory sexual selection by promoting the fertilisation success of an ejaculate through distinct (although not necessarily mutually exclusive) functionalities. First, some SFPs can have a direct influence on the fertilising performance of self-sperm by modulating sperm motility and successful sperm storage within the female sperm storage organs. For example, mammalian SFPs may increase sperm velocity by accelerating the conversion of glucose into ATP^3^. In *Drosophila melanogaster*, the SFP Acp36DE facilitates post-copulatory sperm storage. Females mated to Acp36DE mutant males store only 15% of sperm and produce 10% of progeny relative to those mated to wild-type males^4^. Not surprisingly, Acp36DE mutant males are disadvantaged in sperm competition when competing against wild-type males with normal expression of Acp36DE^5^. Second, SFPs may have ‘offensive’ effects, by reducing the fertilising efficiency of competing (i.e. non-self) ejaculates^6^, or ‘defensive’ effects which reduce the future risk of sperm competition, for example through the formation of mating plugs^7^. These ‘defensive’ effects can also be achieved by manipulating female responses to the male’s advantage – e.g. by reducing female propensity to remate or accelerating female oviposition rate. Amongst *D. melanogaster* SFPs, Sex Peptide (Acp70A) delays female remating^8^, while ovulin (Acp26Aa) enhances short term egg production by the female^9^. These functionalities, while adaptive to males within the context of postcopulatory sexual selection, can also have detrimental repercussions on the fitness of females, and have been put forth as potential examples of sexual conflict^10^.

Consistent with their role in sexual selection, SFP genes often experience accelerated rates of adaptive evolution. For example, the *SEMG2* gene has experienced accelerated evolution in polyandrous species of primates in relation to monandrous species. This gene encodes a predominant component of the copulatory plug, a trait crucial to the fertilisation success of competing males participating in subsequent copulations^11^. Similarly, the molecular mass of the SFP SVSII, which is responsible for the formation of the copulatory plug in muroid rodents, correlates positively with the level of polyandry across species^12^, and the gene encoding this protein is subjected to positive selection in lineages with higher levels of sperm competition^13^. Thus, polyandry can drive the rapid evolution of SFPs and this, in turn, can contribute to the establishment of reproductive barriers and, eventually, to speciation^14^,^15^.

However, the study of SFPs remains taxonomically restricted and largely limited to populations that have long adapted to captivity or domestication. In particular, little is known about SFPs and their functional significance in wild birds (but see ref. 16), despite the fact that birds have long served as a paragon of sexual selection and more recent evidence that supports the influence of SF on reproductive fitness^17^,^18^. Avian SFP biology also has the potential to be unique because birds lack accessory tissues dedicated to the production of SFPs, such as the seminal vesicle in mammals and accessory gland present in some insects. Here, we begin to address this gap in knowledge by characterizing the SF proteome of a population of Red junglefowl (RJF), *Gallus gallus ssp*. The RJF is an appropriate model for the study of avian SF for the following reasons. First, RJF populations are typically polyandrous and the mechanisms of pre- and post copulatory sexual selection are reasonably well understood^19^,^20^,^21^. Second, because the RJF is the wild species that gave rise to the domestic chicken, *G. domesticus*^22^, information on the physiology of chicken SF can be used to infer the functional significance of the RJF SF proteome, and, in turn, the RJF proteome can shed light on the influence of artificial selection associated with domestication. Domestication has had a profound impact on chicken reproductive biology^23^, and information on domestic SFPs^24^ provides a unique opportunity to investigate the molecular basis of reproductive divergence between domesticated and undomesticated species.

Physiological studies of domestic chickens indicate that the SF of ejaculates consists of two components: seminal plasma and transparent fluid. Seminal plasma is produced by the testis and vas deferens, and sperm are expected to be continuously exposed to these secretions as they leave the epididymis. Transparent fluid, a plasma-like secretion produced from the tumescent lymphatic folds surrounding the cloaca, has a similar composition to a dialysate of blood plasma, and is mixed with sperm upon ejaculation^25^,^26^. Empirical evidence from domestic chickens indicates that SF modulates aspects of sperm motility and fertilising efficiency^23^,^26^,^27^, although the molecular nature of these effects remains unclear^23^. The role of SF in sperm motility is particularly significant, given that swimming velocity is a major determinant of fertilisation success under sperm competition^28^,^29^. Importantly, sperm velocity tends to decline as individual males age past their prime^30^,^31^, and evidence from RJF populations suggests that this is mediated by changes in the antioxidant capacity of SF^32^. Collectively, these data suggest that SFPs may modulate aspects of male fertilising efficiency and reproductive ageing.

The aims of this study were to: (i) systematically characterize the SF proteome of RJF; (ii) identify proteome characteristics associated with male age and/or sperm swimming velocity; and (iii) compare and contrast these data with published SFP data in domestic chickens.

## Results

### The Red junglefowl SF proteome

**Figure 1:**
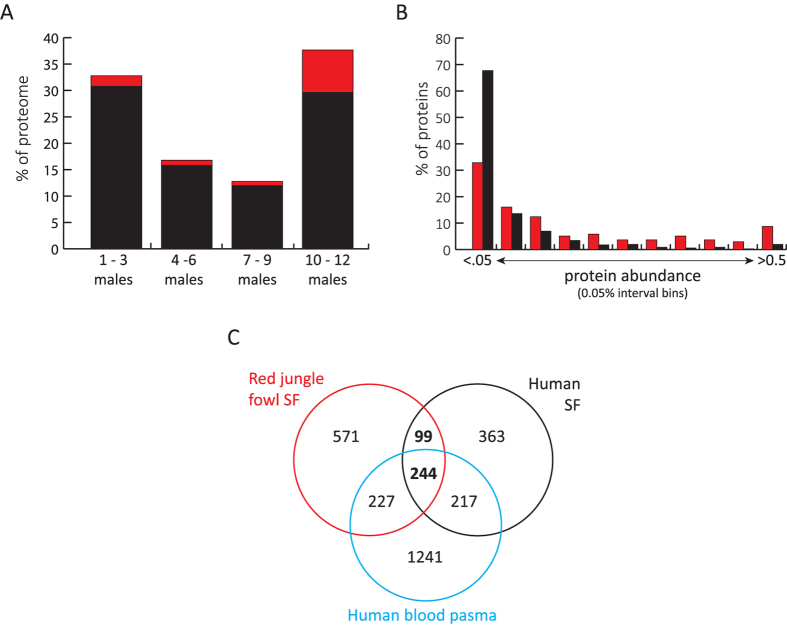
Red junglefowl seminal fluid protein identification and composition. (**A**) Histogram displaying the proportion of the seminal fluid proteome identified as a function of the number of males in which proteins were identified. Proteins also identified as components of blood plasma are indicated in red. (**B**) Histogram comparing the distribution of protein abundance amongst seminal fluid proteins identified (red) and not identified (black) as components of blood plasma. (**C**) Venn diagram displaying the protein overlap between Red junglefowl SF (red), human SF (black) and human blood plasma (blue). The vast majority of conserved SF proteins are also present in the blood plasma proteome.

Tandem mass spectrometry analyses were conducted on SF from 12 male RJF, where each sample was the product of 3 pooled SF samples from the same male. Analysis of 144 protein fractions in total resulted in 200,622 high-quality spectral matches to peptides and the identification of 1,141 proteins (Table S1, Supporting Information). Proteins were required to be identified by no fewer than 2 unique peptides and of these proteins, 24.8% were identified in all 12 males and 55.9% were identified in at least half of the males surveyed (Fig. 1a). The proteomic complexity of RJF SF is therefore much greater than those in other well-studied invertebrates, such as *Drosophila* and mosquito, *Aedes aegypti*, where 138 and 93 ejaculated SFPs have been identified, respectively^33^,^34^ but comparable in size to the SF proteome in humans^35^.

To examine the contribution of transparent fluid to the RJF SF proteome, we investigated overlap with existing proteomic data from domestic chicken blood plasma^36^. This revealed that 52.1% (137 out of 263) of identified blood plasma proteins were present in RJF SF (Table S1, Supporting Information). These proteins were more commonly identified across most or all SF samples analysed than the remainder of the SF proteome (Fig. 1a) and a substantial number of these (46.7%; 64 out of 137) are amongst the most abundant 20% of SFPs. Gene ontology analysis of SF/blood plasma proteins identified numerous biological process enrichments (Table S1, Supporting Information), including response to stress (3.2E-19), defense response (3.26E-16), hemostasis (8.1E-12), regulation of peptidase activity (1.1E-12) and immune system process (8.4E-14). Predominant components of SF and blood plasma include serum albumin, transthyretin and ovotransferrin. Quantitative analysis indicated that 29% of SFPs by mass are accounted for by proteins also found in blood plasma and that on average these proteins are significantly greater in abundance than the non-blood plasma components of the SF proteome (*p* < 0.0001; Fig. 1b). These observations confirm that plasma secretions within the male reproductive track are a major component of RJF SF and likely represent the contribution of transparent fluid. To assess if these abundant SF components are evolutionarily conserved blood plasma proteins, we then compared our proteome to that of human blood plasma^37^. This revealed that the vast majority of SF proteins in chicken blood plasma (122 of 134) were present in the human plasma proteome and identified an additional 349 RJF SF proteins present in human blood plasma that have yet to be identified in the chicken plasma proteome. We note that the proteomic coverage of human blood, compiled from 91 high-quality MS/MS datasets, far exceeds that in chicken. Based on this observation it was then logical to determine if the prevalence of blood plasma proteins in the RJF might be a common feature of vertebrate ejaculates. A comparison of our SF proteome to that of humans, a dataset that was generated in a similar fashion using SF purified from whole ejaculates^35^, identified 343 proteins in common, including 244 (71.1%) also present in the blood plasma proteome (Fig. 1c). Therefore, blood plasma proteins comprise a large fraction of SF proteins identified in whole ejaculates of RJF and humans, and accounts for most of the proteins identified in both species.

A diverse repertoire of immunity proteins function in reproduction and several impact fertility through direct or indirect influences on sperm^38^. A survey of the SF proteome revealed a wide array of molecules which function in immunity, such as (i) innate immunity proteins, including 10 proteins in the classical complement pathways (Table 1), (ii) a diverse set of adaptive immunity proteins, including all four annotated chicken immunoglobulin proteins, and (iii) the avian antimicrobial proteins gallinacin 9 and 10, which are highly abundant and comprise 0.73 and 0.93% of the total protein in SF, respectively (Table 2). It is noteworthy that several abundant immunity proteins are absent from the domestic chicken SF proteome (see below).

### SF proteome and post-spermatogenic sperm maturation

Fowl sperm do not complete maturation in the epididymis^39^ and, consistent with the idea of continued maturation in the vas deferens, research in domestic chickens has shown that sperm from the testis, epididymis, and three segments of the vas deferens experience a progressive increase in survival, fertilising ability, motility, acrosomal proteolytic activity, sperm penetration of the egg perivitelline layer, and binding capacity to the uterovaginal junction sperm storage tubules and infundibulum^40^. To explore the possibility that SF (or secreted vesicles therein; see below) might contribute to extragonadal maturation, we assessed the overlap between sperm proteins previously identified in a population of domestic broiler chickens^24^ and two independent RJF SF datasets derived from SF purified using distinct protocols to control for potential sperm contamination (see Methods). The first dataset represents the combined proteome of 12 RJF samples described above, of which 9.55% were also specifically identified in domestic chicken sperm (Table S1, Supporting Information). The second dataset, which included SF samples derived from 4 independent ejaculates from a single male, exhibited an almost identical overlap with sperm proteins (9.78%). Importantly, 78.9% of these SFPs also identified in sperm were present in both of our SF samples, strongly supporting a relationship between SF and sperm composition. This pattern is also evident in ref. 24, which identified a substantial number of proteins (n = 234) common to both domestic chicken SF and sperm. A survey of these 234 proteins revealed that 92.3% and 94.4% of these proteins were also identified in our RJF SF datasets, respectively. We then asked if these predicated sperm proteins identified in our SF dataset represent evolutionary conserved sperm components present in human^41^ and mouse^42^ sperm. Of the sperm proteins present in our dataset, 81 (74%) and 68 (62%) were also present in the human and mouse sperm proteomes, respectively, further supporting the potential importance of these proteins in sperm maturation and function.

Gene ontology analysis of SFPs also identified in chicken sperm further suggests a predicted role for SFPs in flagellum development. A significant enrichment of cytoskeletal (1.0E-22), microtubule (5.6E-15), cilia (2.5E-6) and flagella (7.2E-5) proteins was observed, including subunits of chaperonin containing TCP-1 complex, which is responsible for actin and tubulin folding. Enrichment of protein complex subunits involved in axoneme elongation were also identified, including proteasome core complex, alpha-subunit complex (1.3E-9) and microtubule organizing centre (5.1E-6). The identification of 12 flagellum proteins amongst the 284 protein identified in all 12 males (4.2%) exceeds the proportion of mouse (1 out of 69)^43^ and *Drosophila* (3 out of 138)^33^ SFPs with predicted subcellular localization in the flagellum. Although we cannot entirely rule out the possibility of sperm protein contamination during SF purification, the highly reproducible results obtained using alternative purification techniques supports the presence of integral sperm components in RJF SF.

### Proteomic support for SF exosomes

Sperm modification after spermatogenesis has been well documented in mammals, where sperm undergo complex modifications within the epididymis^44^,^45^. This process is governed, in part, by extracellular vesicles, termed epididymosomes, which are believed to deliver molecular components to sperm^46^. To explore the possibility of a similar mechanism in RJF, we investigated the presence of the most common protein markers of exosomes^47^ within our SF dataset. This revealed the presence of 85.6% (60 out of 70) of the top exosome markers with one-to-one orthology between chickens and mammals (Table S1, Supporting Information). This included 7 members of the ras-related Rab protein family, which regulate vesicle formation, trafficking and membrane fusion, 6 annexin proteins, which are membrane scaffolding proteins that regulate vesicle formation, and 6 members of the 14-3-3 protein family, which regulate vesicle targeting through cytoskeleton interactions. Exosome markers in SF were also found to be significantly more abundant on average than the remainder of the proteome (non-parametric Kolmogorov-Smirnov test; D = 0.523; *p* < 0.0001), suggesting that exosome-like vesicles may be abundant in SF.

### Proteomic changes associated with male age and sperm velocity

**Figure 2:**
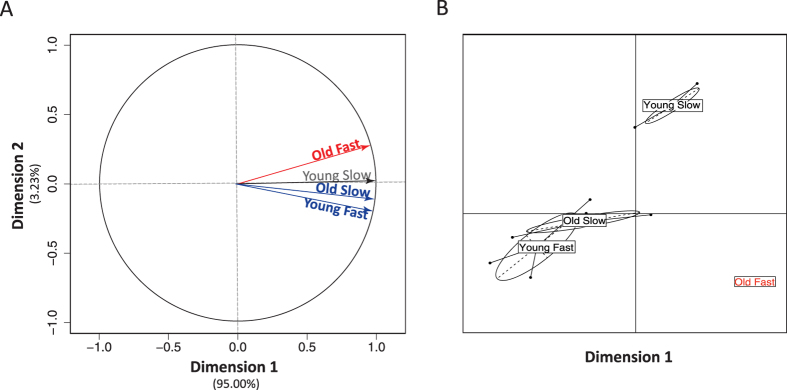
Multivariate ordination of seminal fluid protein abundance with respect to male age and sperm velocity. (**A**) Principle Component Analysis (PCA1) variables factor map. The direction of the arrows represents the relative loadings of the four categories (Old Fast, Old Slow, Young Fast, Young Slow) on the first and second dimension. All four categories have strong positive loadings on dimension 1; Old Fast has a significant positive loading and Old Slow and Young Fast have significant negative loadings on dimension 2, respectively. (**B**) Between group PCA (PCA2) biplot. First and second dimension loadings for the four categories of males are displayed, as well as the extent of within category variance (ovals) and loadings of individuals within each category (points at the end of solid lines).

We first performed PCA1 to summarise the variance in protein abundances across the four categories (Young Fast, Young Slow, Old Fast and Old Slow). The first dimension explained 95% of the variance in protein abundance across categories (Fig. 2a). The four categories loaded strongly with this dimension, indicating that it captures patterns of protein abundance variation that are consistent across the four categories (Fig. 2a; z-transformed protein abundance yields qualitatively identical results (data not shown)). Thus, dimension 1 captures the dynamic range of relative protein abundance within samples rather than absolute changes across samples. The values of the squared cosine for dimension 1 reflect the major contribution of abundant proteins to this dimension since the 30 most abundant proteins in the dataset have squared cosines scores between 0.83 and 1. The five proteins with the highest weighting on dimension 1 (i.e. the most abundant) were: serum albumin precursor, transthyretin precursor, fatty acid-binding protein brain, pendrin isoform X2, and gallinacin-10 preproprotein; collectively they explain more than 42% in the construction of dimension 1 (Table S2, Supporting Information). Conversely, for example, amylase (alpha 2A) is low in abundance and one of the lowest coordinates. Dimension 2 of PCA1 explained 3.23% of the variance in protein abundance across categories (Fig. 2a). Old Fast was the only category that had a significant positive loading with dimension 2, while Old Slow and Young Fast both had significant negative loadings (Table S3, Supporting Information), indicating that this dimension may reflect protein abundance variation that is distinct in the ejaculates of old males with fast sperm (Fig. 2a). The between-category PCA2 confirmed this pattern, revealing that Old Fast samples were tightly clustered together and away from the other categories in dimension 1, with clear separation from Old Slow and Young Fast (Fig. 2b). We also note that Young Slow samples occupy a unique space, particularly in relation to dimension 2, but do not cluster as tightly as Old Fast males. Finally, the NMDS analysis summarized the variance in a 2-dimensional ordination space with high fit with the original protein ranks (stress = 0.116; Table S3, Supporting Information). Once again, the Old Fast category emerged as different from the other three categories (NMDS dimension 1; Figure S3, Supplemental Information), confirming the results of PCA1 and PCA2.

**Figure 3:**
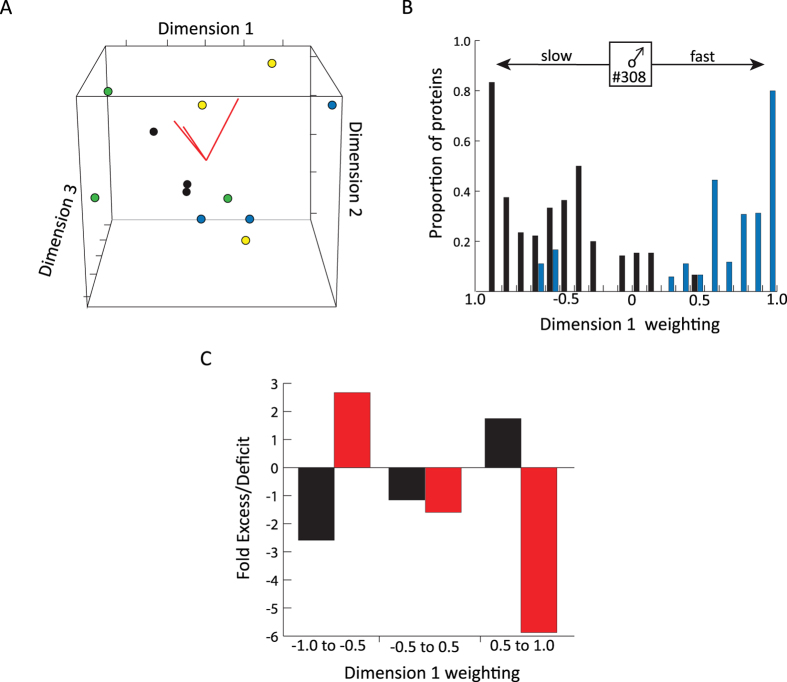
Seminal fluid protein abundance variation associated with sperm velocity in old males. (**A**) 3-dimension visualization of Principle Component Analysis (PCA3), using the inclusive protein dataset as variables (Old Fast, blue; Old Slow; black; Young Fast; green; Young Slow, yellow). Dimension 1 captures variance associated with old male sperm velocity, including negative weightings for Old Slow and positive weightings for Old Fast. (**B**) Histogram displaying the distribution of first dimension weightings for proteins exhibiting significant abundance differences between the Slow and Fast sample of the replicated old male (#308)(abundance increase in fast sample, blue; abundance increase in slow sample, black). (**C**) Histogram displaying the excess or deficit of blood plasma proteins (red) and exosome markers (black) across first dimension weight ranges.

PCA3 summarized variation in the profile of individual SFPs across the 12 samples. This analysis identified 11 dimensions, of which the first 3 explained 51.2% of the cumulative variance (Table S3, Supporting Information). Importantly, dimension 1 (eigen value = 219.56, 26.14% of variance explained) captures a gradient in sperm velocity, discriminating Old Slow from Old Fast samples (Fig. 3a). Consistent with this, two of the three old slow males occupy negative coordinates in dimension 1 while all three old fast males occupy positive coordinates in this dimension (Table S2, Supporting Information). We next analyzed dimension 1 weightings of proteins exhibiting significant abundance differences within the single replicated old male (#308) who was sampled with both Fast and Slow sperm velocity. This revealed a striking association between the distribution of dimension 1 weightings and directional changes in protein abundance between Fast and Slow samples from this male (Fig. 3b). To explore the functional basis for dimension 1 weighting associations, we compared the weightings of blood plasma and exosome marker proteins identified above. This revealed a strong disparity between these functional classes, including (i) an enrichment for blood plasma proteins on the negative side of this dimension (Old Slow), (ii) a deficit of blood plasma protein on the positive side (Old Fast) and (iii) the inverse pattern for exosome-related components (Fig. 3c). Consistent with this observation, a substantial number of blood plasma proteins were significantly more abundant in the Slow sample of the replicated Old male (#308) relative to his Fast sample. Interestingly, a somewhat opposite pattern for differentially abundant blood plasma proteins is observed for the young male (#H28) sampled twice (i.e. both Slow and Fast sperm velocity; data not shown). Consistent with the colocalization of Old Fast and Young Slow on dimension 1, 26 of 43 blood plasma proteins are more abundant in the H28 Fast sample. Interestingly, these proteins are largely distinct from the blood plasma proteins identified as differentially abundant in male #308. Taken together, these observations suggest that variation in sperm velocity across males is determined by the abundance of blood plasma and exosome proteins in SF. The detection of distinct sets of proteins in the young and old males outlined above further suggests that the proteins and molecular pathways governing sperm velocity may be age-specific.

Two-way ANOVAs of individual proteins identified 9 cases in which, after controlling for false discovery rates, a protein was differentially expressed with respect to male age, sperm velocity or their interaction (Table 3; top section). Of these 9 proteins, five are proteins more abundant in Old Fast samples. Interestingly, patterns of differential protein expression associated with sperm velocity across males were recapitulated in changes within the two males that were replicated twice and represented in both the Slow and Fast categories. This included three proteins up-regulated in the Old Fast category (fibulin 2, proto-oncogene protein kinase ROS and receptor-type tyrosine phosphatase eta precursor) and two proteins exhibiting differential abundance between the Young Fast and Young Slow categories (beta-galactoside-binding lecting and inosine-5′-monophosphate dehydrongenase 2). A total of 12 proteins were also identified exclusively and consistently amongst males of a single age (1), velocity (1) or age-velocity category (10) (Table 3; bottom section). Of these, 8 were found in the category Old Fast, which is consistent with the distinct nature of this category as identified by the PCA and NMDS analyses (above). The number of proteins identified exclusively in Old Fast males is unique within the dataset, as the identification of multiple proteins in a specific subset of 3 males was otherwise rare. Lastly, we re-ran PCA1 of the ‘inclusive’ proteome (above) excluding those proteins identified here by ANOVA and restricted to specific age-velocity categories. This analysis yielded nearly identical results, indicating that the loading of Old Fast samples on dimension 2 of PCA1 is not solely driven by a subset of SFPs, but reflects a general property of SF proteome profiles.

### Comparative analysis of the RJF and domestic chicken SF proteome

**Figure 4:**
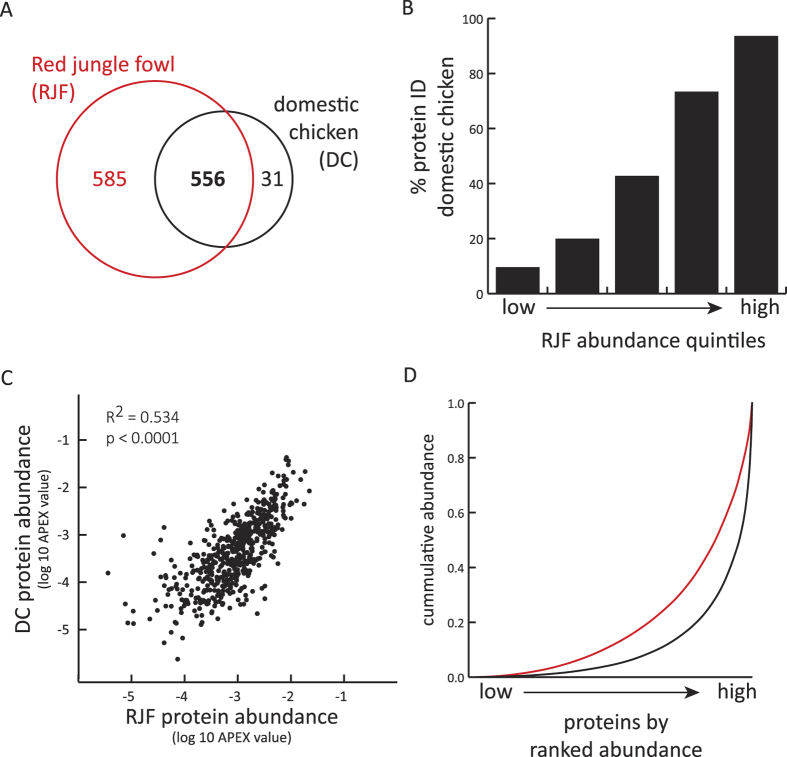
Comparison of the Red junglefowl and domestic chicken seminal fluid proteomes. (**A**) Venn diagram indicating the size and overlap between the seminal fluid proteomes of the Red junglefowl and domestic chicken. (**B**) Overlap in proteins identified in the domestic chicken in relation to protein abundance in the Red junglefowl proteome. (**C**) Linear regression identifies a significant correlation in the abundance of proteins identified in both the domestic chicken and Red junglefowl proteomes. Protein abundance is plotted as log transformed APEX values, which represent normalized estimates of protein abundance in each sample. (**D**) Cumulative protein abundance distribution of the domestic chicken (black) and Red junglefowl (red) proteomes.

A direct proteomic comparison, based on the reanalysis of raw MS/MS data from domestic chicken SF samples, revealed that 94.4% of domestic SFPs were identified in our RJF proteome. 31 proteins were identified as unique to the domestic SF proteome (Table S4, Supporting Information), while 585 RJF proteins were absent from the domestic proteome (Fig. 4a). Domestic SFP identification was significantly enriched amongst more abundant RJF proteins (Χ^2^ = 459; *p* < 0.0001), indicative of limited coverage of low abundance proteins in the domestic chicken (Fig. 4b). Given the more comprehensive nature of the RJF proteome, proteins uniquely identified in the domestic chicken are likely to represent *bona fide* SF proteome differences between domestic chickens and RJF. Notable amongst these 31 proteins are a significant enrichment in cell recognition proteins (e.g. Cadm1 and Arsa). The relevance of these proteins to fertility is indicated by mammalian phenotypes, including the role of Arsa as an acrosome vesicle zona pellucida binding protein and sperm maturation defects and infertility in Cadm1 mutants. Gst3, a detoxifying enzyme and potential mediator of ROS damage to sperm, was also identified uniquely in the domestic chicken. The substantial overlap (>93%) between the RJF and domestic chicken proteomes amongst the most abundant protein class (Fig. 4b) supports the possibility that some highly abundant proteins not identified in the domestic chicken may be RJF-specific (Table S4, Supporting Information). Gene Ontology analyses of predicted RJF-specific proteins reveals a significant enrichment of proteins predicted to be involved in humoral immunity (3.6E-4) and complement activation (5.7E-3), amongst a diverse set of immunological process categories. Complement factor H, C8 gamma and immunoglobulin mu chain are of particular note as putative RJF-specific SFPs given their high abundance and consistent identification across individuals. Epididymal secretory protein E1, which has been implicated in mammalian sperm maturation, is also noteworthy.

A significant correlation in protein abundance was observed amongst the 556 proteins identified in both the domestic chicken and RJF proteomes (Fig. 4c), despite the systematic differences in protein abundance estimates revealed by comparing cumulative abundance distributions (Fig. 4d). Direct abundance comparisons would therefore be predicted to result in many low-to-moderate abundance proteins appearing to be of greater abundance in RJF and a smaller set of highly biased proteins as greater in abundance in the domestic chicken. To minimize this issue, an approach was implemented that assessed the shift in both a protein’s abundance rank and abundance estimate between species. This analysis successfully identified quantitative differences in a largely unbiased bidirectional pattern between taxa, including proteins with coherent functions (Table S4, Supporting Information). A significant enrichment was confirmed in the following biological processes: defense response (1.6E-4), response to stress (3.1E-4), inflammatory response (3.8E-3) and regulation of immune system process (3.9E-2). Three of these proteins (IgM, IgG and IgGFc-binding protein) were found to be quantitatively more abundant in RJF than domestic chicken, consistent with the identification of other immunity proteins putatively specific to the RJF SF proteome.

## Discussion

This study presents a systematic SF proteome analysis of a non-domestic, polyandrous bird population and one of the first investigations of the signatures of male age and sperm quality in a SF proteome across a population. While the functions of SFPs have received substantial interest^2^, much of this work has focused on a few lab-adapted or domestic study organisms. Information on SFPs is particularly patchy in birds, despite the importance of this group to the study of sexual selection. Information on avian SFPs is largely restricted to a recent study of a domestic broiler chicken population^24^. Our study contextualizes this earlier work, by conducting a more comprehensive analysis of a population that has not been exposed to artificial selection. Below we discuss the key aspects of: (i) the composition of the RJF SF proteome, (ii) SF proteome age and sperm velocity-dependent variation, and (iii) the signature of domestication and artificial selection.

In mammals and insects, specific reproductive tissues are responsible for the production of SFPs. Analogous dedicated tissues have yet to be identified in birds and many birds, such as passerines, produce little SF^26^. Fowl, on the other hand, produce substantial quantities of SF^23^. Our comparative analysis of the SF and blood plasma proteomes reveals that plasma-like secretions represent a major component of both fowl and human SF. Although the functional significance of this transparent fluid in fowls remains unclear, it may contain SFPs that contribute to ROS buffering and the protection of sperm from oxidative damage during prolonged periods of sperm storage in the male extragonadal reserves and in the female storage tubules. The present study also indicates that RJF SF proteome has a compositional complexity that exceeds that of insects and is likely to be more comparable to that of the characterized human SF proteome^35^, indicative of diverse range of functions (see below). The substantive contribution of broadly expressed plasma proteins (with putative “housekeeping” roles), appears to stand in stark contrast to the highly specialized repertoire of male-specific SFPs in other species. However, our understanding of gene expression across both male and female reproductive tissues in birds is very limited. Additional proteomic and transcriptomic studies will be required to determine if avian SF also contains highly specialized, male-biased reproductive proteins and, if so, where they originate from in the male reproductive tract.

A diversity of innate and adaptive immunity proteins, as well as antimicrobial proteins, were identified in RJF SF. Immunity proteins are widespread in both male and female reproductive systems^38^ and some SFPs are known to target microbes that pose a threat to sperm viability and clear pathogens introduced during copulation, thus protecting the egg from potential infections^48^,^49^,^50^,^51^,^52^. Evidence suggests that avian semen can harbour bacteria^53^,^54^ and that the SF of some birds may have antibacterial properties^6^,^18^,^52^. Our results confirm that antimicrobial proteins gallinacin 9 and 10, of the defensin protein family, are amongst the most abundant proteins in the RJF SF proteome, a finding consistent with SF analysis in domestic chickens^24^ and of particular relevance for promiscuous populations of RJF, where ejaculates host diverse bacterial communities (R. Eccleston & T. Pizzari, unpublished). Immunological proteins may also play a key role in genetic recognition, such as kin-biased interactions between ejaculates of different males or interactions between semen and the female reproductive tract. For example, SF can affect sperm fertilising efficiency differentially based on the genetic similarity between sperm and SF donor males in some taxa (e.g. refs 6 and 55). Polyandrous RJF females may also select against sperm of closely related males following mating, a response that appears to be associated with male-female similarity at the Major Histocompatibility Complex locus (MHC)^56^. Two proteins belonging to the MHC Class I (beta-2-microglobulin, and ATP synthase subunit beta, mitochondrial precursor), and one to the MHC Class II (class II histocompatibility antigen, B-L beta chain) were identified in our study. Both MHC Class I proteins are also present in the domestic chicken SF. Interestingly, the class II histocompatibility antigen, B-L beta chain was not present in domestic chicken SF (see discussion of domestication below). Our proteomic results are consistent with the proposition that MHC variation may provide a molecular cue for post-copulatory genetic recognition between rival ejaculates and between mates.

In many species, sperm are not mature or fertilisation competent following spermatogenesis and there is evidence that fowl sperm may also acquire fertilisation capacity progressively through their passage through the vas deferens^40^,^57^,^58^,^59^,^60^. Consistent with this idea, a comparison of two independently derived RJF SF proteomes with the domestic chicken sperm proteome revealed considerable overlap and a significant enrichment of SFPs that are structural components of the flagellum. An alternative, non-mutually exclusive explanation of this overlap is that sperm proteins are released into SF as sperm cells age and degrade. While the present study cannot explicitly distinguish between these alternatives, a careful inspection of our proteomic data reveals the presence of numerous marker proteins that are commonly associated with exosomes. Exosomes produced in the epididymis of mammals are believed to play a prominent role in post-spermatogenic sperm maturation and their molecular cargo (*e.g*. protein or RNA) have been the focus of investigation^46^. A similar process has also been characterized in *Drosophila*, where exosomes produced in the secondary cells of the accessory gland bind and fuse with sperm^61^. Although it is not possible to make firm conclusions based on our data alone, our analysis is consistent with the continued maturation of sperm during transit, and points to the presence of secreted vesicles that could sustain the final stages of maturation in a manner analogous to those described in other systems. Members of the 14-3-3 protein family, which are diverse exosome markers in our dataset, influence mammalian sperm maturation and motility through their interactions with sperm-specific protein phosphatases^62^. Importantly, 14-3-3 eta and zeta are decreased in abundance in SF of subfertile domestic chickens^24^ and 3 distinct protein phosphatases were identified in our study as differentially abundant based on age and velocity.

Sperm velocity is a key determinant of fertilising efficiency in domestic chickens^39^, particularly under sperm competition when ejaculates with relatively high sperm mobility progressively achieve a competitive edge over female sperm storage time^28^. However, sperm velocity tends to decline as males age^30^,^31^, and these age-dependent effects are associated with changes in SF composition^32^. This study set out to provide the first characterization of the SF proteomic profiles associated with male age and sperm velocity in a wild species of bird. However, we urge caution in the interpretation of our results and stress that additional studies, using complementary quantitative techniques, will ultimately be required to achieve a refined understanding of SF proteomic variation.

Multivariate analyses distinguished the proteome of Old Fast samples from that of the other three categories of males. These results suggest that high sperm velocity in older males is associated with higher abundance of exosome markers, potentially linked to sperm maturation. Eight proteins were identified exclusively in the samples belonging to the three males of this category. Although it would be premature to draw any specific functional conclusions, many of these proteins are involved in calcium metabolism. For example, calmodulin, a calcium-binding messenger that controls sperm activation and hyperactivation in mammals, activates kinases in chicken sperm that influence both viability and motility^63^,^64^. It will be of great interest to determine if and how secreted calmodulin in SF might influence RJF sperm maturation and the acquisition of motility. Additionally, the S100 proteins are involved in calcium homeostasis and can function in concert with various growth factors following extracellular secretion^65^. Interestingly, neuroblast differentiation-associated protein AHNAK-like, also identified exclusively in Old Fast males, is implicated in calcium regulation and constitutes a calcium-dependent S100B target protein^66^. In domestic chickens, calcium constitutes a primary determinant of sperm mobility^67^. Excessive calcium uptake can result in mitochondrial degeneration, as was found to be the case in roosters with low sperm mobility^68^. It is possible that these proteins may ameliorate the decline in sperm velocity associated with ageing, through the mediation of calcium uptake. One possibility is that only some males may be able to retain high rates of sperm maturation in old age. Conversely, semen of Old Slow males (with low rates of sperm maturation) is characterized by relatively high amounts of blood plasma proteins, which likely originate from transparent fluid. These patterns could be due to two alternative mechanisms. First, plasma proteins may cause a reduction in sperm velocity. Second, given that many abundant plasma proteins have either buffering or antioxidant properties, it is tempting to speculate that ageing males with slow rates of sperm maturation may partially ameliorate the fertilizing efficiency of their ejaculates by preferentially investing in transparent fluid. The former mechanism would suggest that in the absence of elevated plasma proteins in their SF, the ejaculates of Old Slow males may display higher sperm velocity while the latter mechanism would predict even lower sperm velocity and/or faster rates of sperm mortality. At present the specific role of transparent fluid in fertilizing efficiency in fowl remains inconclusive^25^, and more research is required to test these hypotheses. In light of the conserved contribution of blood plasma proteins to SF in fowl and humans, it is interesting to speculate that blood plasma protein abundance might also influence sperm quality characteristics across a wide range of vertebrates, possibly including humans.

Genetic homogenization during domestication of the chicken is likely to have resulted from a combination of intense artificial selection, life-history trade-offs and bottlenecks. The impact of this at key genomics regions, such as those important contributions to immunity and fertility^69^, may underlie the well documented reductions in performance amongst domestic chickens and resultant impact on the poultry industry^70^. This loss of function is particularly pronounced in broiler breeders where extreme artificial selection for growth rate has resulted in a trade-off with traits associated with immunity and fertility^4^,^71^. Our comparative results, although preliminary, highlight divergence between the SF proteome of RJF and that of a population of broiler breeders. We note that methodological differences, including the analysis of larger quantities of proteins and substantially greater numbers of MS/MS runs per male in the current study, has resulted in a far greater depth of coverage in the RJF relative to the domestic chicken. Nonetheless, we show that predicted RJF-specific SFPs are functionally coherent, being more likely to be involved in immunological defenses, consistent with a loss of genetic diversity associated with domestication. A number of factors may underpin this selective disappearance in domestic chickens. For example, genes associated with immune responses may have been “lost” during domestication due to a trade-off with genes supporting different functionalities promoted by artificial selection (*e.g*. production traits), possibly catalyzed by the protection of domestic populations through artificial means (*e.g*. vaccines and antibiotics) which may relax selection on immunity genes and pathways^72^,^73^.

The observation that several SFPs associated with male age and sperm velocity have yet to be identified in the domestic chicken SF proteome is also consistent with the notion that domestication may have led to reductions in male fertility through changes in SF composition. While morphological and behavioural traits, as well as sperm traits, have been evoked to explain reduced male broiler breeder fertility, the results of the present study indicate that SFPs may also play a key role. Additionally, in populations under natural conditions, such as in our study RJF population, males are sexually selected throughout their lifespan, which often extends past their prime^74^. Genes that encode SFPs conferring fertilisation efficiency late in life will be favoured by postcopulatory sexual selection. In domestic populations, which are typically female-biased and where males are culled early in life, sexual selection on late life fertilising efficiency will be relaxed, favoring the evolution of early reproductive senescence. Therefore, our study creates a platform for the identification of SFPs “swept away” by the domestication process, some of which might explain fertility deficits in domesticated chickens. Given that our study was based on semen samples obtained through manual abdominal massage, a related area of future research will be to establish the way in which individual males modulate allocation of different SFPs across natural ejaculates, which are notoriously plastic due to strategic ejaculate expenditure in response to multiple socio-sexual factors^75^.

## Materials and Methods

### Semen collection and SF isolation

We studied a population of RJF at the John Krebs Field Station (Oxfordshire), which was founded in 2006 from other captive populations. All sampling of birds was conducted in accordance with approved experimental protocols under Home Office licence (PPL 30/2931). In July-September 2013 semen samples were obtained from 16 sexually mature males by abdominal massage, which captures a representative sample of extragonadal semen reserves of a male^23^. Males were isolated from females for 48 hours before being massaged for the first time. Two age classes of males were selected: “Young” (1 year old, n = 7), and “Old” (4, 5, and 7 years old, n = 9), which collectively represent a relevant dataset to detect age-dependent declines in fertility^30^,^76^. Males were kept sexually rested and isolated from females for 48 h. For each male, semen samples were collected every four days to allow full replenishment of extragonadal sperm reserves, between 12:00–17:00 h. In total, 157 semen samples were collected (77 from young, and 80 from old males). Immediately following collection, samples were placed in a water bath at RJF physiological temperature (41C). We then diluted 1 μl of semen into a culture medium containing 180 μl of Dulbecco’s Modified Eagle’s Medium (DMEM) and 20 μl of Standard Foetal Bovine Serum (FBS). We transferred 20 μl of this mixture to a microscope slide placed on a heated microscope stage set at 41C and connected to a digital video camera set at 10x magnification, and measured sperm average path velocity (VAP) using the Sperm Class Analyzer: SCA v. 3.0.3 (Microptics) set for chicken with the following specifications: capture 25 images/sec; tracking objects between 50 and 200 microns; Optics: Ph-; Chamber: Leja 10; Scale: 10x. A minimum of 200 individual sperm cells were tracked for each sample, across at least three different fields.

Immediately following VAP measurement, we used the remainder of a sample to separate sperm from SF by centrifugation at 3,500 g for 1 minute at 4C. The SF supernatant was transferred to a sterile tube for storage at −20C. This protocol was designed to purify SF from sperm cells, while reducing damage (i.e. flagellum or midpiece separation) and protein contamination from damaged cells. To conduct an independent assessment of the presence of integral sperm components in SF in samples in which whole sperm were confirmed to be absent by microscopy, we purified semen samples from four separate ejaculates of a single male using a more extensive centrifugation protocol. This included centrifugation at 600 g for 10 minutes at 4C, and, then, centrifuging the supernatant twice more at 600 g for 10 minutes and 3,000 g for 30 minutes at 4C^24^. The absence of sperm cells and flagella in this SF samples was confirmed by phase contrast microscopy. Mass spectrometry analyses of all samples were conducted using the same methodology (see below).

To determine SF proteomic profiles associated with male age and sperm VAP, we created four categories: young males with high VAP (Young Fast), young males with low VAP (Young Slow), old males with high VAP (Old Fast), and old males with low VAP (Old Slow). To do so, we measured the average VAP of each male monthly (Fig. S5a, Supporting Information), and selected the three samples with the highest VAP for each of the three young males with the highest average monthly VAP, and the three samples with the lowest VAP for each of the three young males with the lowest average monthly VAP. We performed a similar selection among the old males. Two males were represented in two different categories (Old Fast - Old Slow, and Young Fast - Young Slow, respectively) as they produced highest and lowest average VAP in two separate months. Pearson correlations within these two males that were replicated twice and represented in both the Slow and Fast categories, indicated that these males had a high overall consistency in their SF proteomic profile (correlation coefficients of 0.85 for the Old male, and 0.84 for the Young male). Therefore, we worked on a dataset of 12 samples from 10 males. This selection resulted in significant differences in VAP across the Young and Old, and the Slow and Fast categories (Fig. S5b, Supporting Information).

### Protein preparation and 1-dimensional SDS-PAGE

Solubilization buffer (4% Chaps, 7 M Urea, and 2 M Thiourea) was added to each SF sample and protein concentrations were quantified using the Quick Start Bradford assay and a spectraMax M2 microplate reader. Bovine Serum Albumin (BSA) was used as a standard (scale from 0–2 mg/ml) and samples were sonicated using a bioruptor to disrupt residual protein interactions. To account for variation between ejaculates, 15 μg of protein were combined from each of three ejaculates per male and diluted appropriately to obtain equal protein concentrations across samples from each of the 12 samples. 45 mg of total protein from each sample was separated for the maximum duration possible (without running small proteins off the gel) on 4–12% Nupage Bis-Tris gels running on an XCell SureLock Mini-Cell PowerEase 200 system (Life-Technologies). Gels were then fixed in 45% methanol and 1.0% acetic acid for 1 h and immediately stained with Coomassie (0.1%w/v Coomassie, 34% methanol, 17%w/v ammonium sulfate, and 0.5% acetic acid). Gels were transferred to a gel slicer where each lane was cut horizontally into 12 slices, resulting in total of 144 samples for MS/MS analysis. To reduce spectra derived from the highly abundant SFP serum albumin and increase proteomic coverage, a narrow region centered on the predominant serum albumin band was not included amongst the fractionated gel slices. Gel slices were subjected to proteolytic digestion using an Automated Preparation Station (Perkin Elmer). Proteins were first reduced (1 hr at 37 °C in 15.4 mg dithiothreitol in 10 ml 100 mM ammonium bicarbonate solution) and alkylated (1 hr at room temperature in 102 mg iodoacetamide in 10 ml 100 mM ammonium bicarbonate solution) followed by digestion with trypsin (500 ng/μl, Promega) at 37C for 16 h.

### Tandem mass spectrometry analysis

Tandem mass spectrometry (MS/MS) experiments were performed using a Dionex Ultimate 3000 RSLC nanoUPLC (Thermo Fisher Scientific Inc, Waltham, MA, USA) system and a QExactive Orbitrap mass spectrometer (Thermo Fisher Scientific Inc, Waltham, MA, USA). Separation of peptides was performed by reverse-phase chromatography at a flow rate of 300 nL/min and a Thermo Scientific reverse-phase nano Easy-spray column (Thermo Scientific PepMap C18, 2 μm particle size, 100A pore size, 75 μm i.d. × 50 cm length). Peptides were loaded onto a pre-column (Thermo Scientific PepMap 100 C18, 5 μm particle size, 100A pore size, 300μm i.d. × 5 mm length) from the Ultimate 3000 autosampler with 0.1% formic acid for 3 minutes at a flow rate of 10 μL/min. After this period, the column valve was switched to allow elution of peptides from the pre-column onto the analytical column. Solvent A was water +0.1% formic acid and solvent B was 80% acetonitrile, 20% water +0.1% formic acid. The linear gradient employed was 2–40% B in 30 minutes. The LC eluant was sprayed into the mass spectrometer by means of an Easy-spray source (Thermo Fisher Scientific Inc.). All *m*/*z* values of eluting ions were measured in an Orbitrap mass analyzer, set at a resolution of 70000. Data dependent scans (Top 20) were employed to automatically isolate and generate fragment ions by higher energy collisional dissociation (HCD) in the quadrupole mass analyser and measurement of the resulting fragment ions was performed in the Orbitrap analyser, set at a resolution of 17500. Peptide ions with charge states of 2+ and above were selected for fragmentation. Post-run, the data was processed and peak-lists generated using Protein Discoverer (version 1.3, ThermoFisher). The mass spectrometry proteomics data have been deposited to the ProteomeXchange Consortium http://proteomecentral.proteomexchange.org) via the PRIDE partner repository with the dataset identifier PXD00005160.

### Peptide identification and protein annotation

Raw data from each tandem mass spectrometry (MS/MS) run was analyzed by X!Tandem^77^ and Comet^78^ against the complete *Gallus gallus* refseq protein database (June 2013 release, 32134 proteins), supplemented with characterized, but yet to be annotated, immunoglobulin sequences. A fragment ion mass tolerance of 0.40 Da and a parent ion tolerance of 10.0 PPM were used. Iodoacetamide derivative of cysteine was specified as a fixed modification, whereas oxidation of methionine was specified as a variable modification. Peptides were allowed up to two missed trypsin cleavage sites. All downstream analyses were conducted using the Trans-Proteomic Pipeline (TPP v4.7 POLAR VORTEX rev 1^79^). False Discovery Rates (FDRs) were estimated with a randomized decoy database using PeptideProphet, employing accurate mass binning model and the nonparametric negative distribution model. X!Tandem and Comet PeptideProphet results were combined using iProphet, to provide more accurate and conservative peptide identification probabilities. Peptide identifications were accepted if they could be established at greater than 95.0% iProphet probability and protein assignations were accepted if they could be established at greater than 99.0% probability. Proteins that contained identical peptides and could not be differentiated based on MS/MS analysis alone were grouped to satisfy the principles of parsimony. The same analysis pipeline was used to reanalyze available raw MS/MS data from fractionated domestic chicken SF samples^24^. SF proteomes were compared to the chicken blood plasma proteome^36^, the exosome ExoCarta high quality protein marker dataset^47^, human blood plasma^37^, human sperm^41^, mouse epididymal sperm^42^ and human seminal plasma^35^ using orthology relationships in Ensembl Genes 84 (Galgal4). Composition of protein datasets were analyzed using the DAVID Bioinformatics Resource (6.7)^80^,^81^, including statistical analyses of functional enrichment using the *G. gallus* genome as the background dataset for comparisons.

### Protein quantitation and analysis

After consideration of serum albumin depletion options and the potential impact of this highly abundant protein on various quantitative approaches, a semi-quantitative spectral counting approach was employed. Protein quantitation was conducted using the APEX Quantitative Proteomics Tool^82^. Fifty SFPs previously identified using the same MS/MS methodology (data not shown) were utilized for the training dataset based on having the highest numbers of spectral counts per protein and the highest protein identification probabilities. The 35 physicochemical properties available in the APEX tool were used for prediction of peptide detection/non-detection in the construction of a training dataset file. Protein probabilities (O_i_) were computed using the Random Forest classifier algorithm trained with the dataset generated in the previous step. APEX protein abundances per male and as a combined dataset were calculated using the protXML file generated by the ProteinProphet. The same approach was used to calculate Apex scores for the domesticated *G. gallus* SF proteome^24^. Given the skew in the distribution of peptide matches for the lower coverage domestic chicken dataset (see Results), proteins exhibiting abundance differences between subspecies were identified as those with the greatest abundance rank shift between proteomes, in conjunction with quantitative differences based on their Apex estimate.

### Analysis of male age and sperm swimming velocity (VAP)

In order to establish SF proteomic profiles associated with male age (Old and Young) and sperm quality (Slow and Fast), we combined multivariate ordinal approaches with the analysis of individual proteins. First, we asked whether variation in the proteomic profile of individual samples could be explained by the sperm velocity and/or the male age of a sample. We therefore summarized variation in APEX-corrected protein spectral counts across the four specified categories (i.e. Old Slow, Old Fast, Young Slow, Young Fast). To do this, Principal Component Analysis, as implemented by the FactoMine R package, was used to compare the protein spectral counts across the four categories (PCA1) after normalization (i.e. equal total number of spectral counts across all males). We focused on an ‘inclusive’ dataset, which included all the proteins (840) identified in a minimum of 3 and up to all 12 samples (a “0” was entered when no spectra were detected for a protein in a given sample). Because each age/velocity category was comprised of 3 males, we chose a minimum of 3 males to identify situations in which a protein was expressed in all samples of a category but nowhere else. For each protein, we calculated the average spectral count for each of the four categories of males. We positioned these categories of males in a variables factor map to visualize their correlations with the two main dimensions of the PCA. To refine the interpretation of the variables factor map, we calculated for each protein over the four dimensions: (a) its coordinates on the dimension; (b) its squared cosine, which measure the quality of the projection of the protein on the dimension; (c) and its contribution (%) to the construction of the dimension. We confirmed these results using between-groups PCA (PCA2)^83^ using the ade4 R package, which distinguishes variance within and across categories. This optimizes group variability, instead of between sample variability, by transforming the abundance data by averaging across the individuals belonging to each age/speed group. The protein and sample weightings, measuring distances between the proteins and samples, respectively, do not change from PCA1. Four groups were used for the PCA2 analysis, representing each of the age by velocity categories. Because PCA can be sensitive to transformation, we verified the robustness of these results with the results from Nonmetric multidimensional scaling (NMDS) in the Vegan R package, using the 840 proteins of the ‘inclusive’ dataset. NMDS is a non-eigenvector technique that summarizes the variance in a N-dimensional ordination space that best represents the differences in protein ranks^84^. Unlike the PCA, NMDS ranks the proteins based on their abundance, therefore eliminating potential problems (e.g., sensitivity to transformation) associated with the use of absolute distances between their spectral counts. NMDS iteratively repositioned the proteins into the ordination space to minimize the disagreement (i.e. stress) between their original distances and their final dimensional representation. We plotted the relationship between the original protein distances and their dissimilarities in the final two-dimensional ordination space using a Shepard stress plot.

Second, we performed a third PCA (PCA3), using the 840 proteins of the ‘inclusive’ dataset, to characterize the 12 samples from the point of view of these proteins (i.e. the spectral count of each SFP is a variable). Thus, PCA3 uses the spectral count of individual protein as variables and summarises this variation across the 12 samples, in a way similar to a PCA of ecological communities in which the abundance of each species (i.e. SFP spectral count) is a variable and the PCA summarises variation in species abundances across a number of sites (i.e. samples). This analysis explores whether any of the main dimensions of the PCA summarizes biologically relevant differences in the proteomics profiles associated with male age and/or sperm quality, by positioning the 12 samples of individual males in the multidimensional space. The rationale of this approach is that, should individuals belonging to a given category have different loadings on a particular dimension than individuals belonging to a different category, the functional analysis of the proteins differentially expressed in these categories will shed light on the biological significance of that dimension. As in PCA1, a “0” was entered when no spectra were detected for a protein in a given sample.

Finally, we complemented the multivariate approach outlined above with an in-depth analysis on each individual protein of the inclusive dataset. To do so, we conducted a two-way ANOVA, with “male age” and “sperm quality” as fixed factors, and “spectral counts” as a response variable. We conducted the Shapiro test for each individual protein. Those proteins that were not normally distributed (p < 0.05) were log 10 transformed (Log_10_(corrected spectral counts + 0.001)). The model fitted in R was: model<-aov (spectral~age*velocity). If the 2-term interaction was not significant, we repeated the procedure by fitting a new model: model1<-aov (spectral~age + velocity). When the ANOVA detected significant effects across the four categories, we performed pair-wise comparisons to determine which categories differed from each other. We used t-tests with a 2-tailed distribution assuming unequal variances (heteroscedasticity) to perform pairwise comparisons of the spectral counts across the 8 possible comparisons, and used the Benjamini-Hochberg formula to control the false discovery rate over the multiple comparisons. This approach identified genes that were differentially up- or down-regulated with respect to male age, sperm velocity or both. In addition, we made use of the fact that two males were represented in two different categories (Old Fast - Old Slow, and Young Fast - Young Slow, respectively). We used Pearson correlations to measure the strength of the relationship of the spectral counts between the two measurements of each male (that is, when belonging to each of the two categories) to quantify overall intra-male consistency in SF proteome, and to identify individual proteins with abundance changes associated with changes in categories that deviated from the overall trend (i.e. proteins with the largest residual).

## Additional Information

**How to cite this article**: Borziak, K. *et al*. The Seminal fluid proteome of the polyandrous Red junglefowl offers insights into the molecular basis of fertility, reproductive ageing and domestication. *Sci. Rep*. **6**, 35864; doi: 10.1038/srep35864 (2016).

**Publisher’s note:** Springer Nature remains neutral with regard to jurisdictional claims in published maps and institutional affiliations.

## Supplementary Material

Supplementary Table S1

Supplementary Table S2

Supplementary Figure S3

Supplementary Table S4

Supplementary Figure S5

## Figures and Tables

**Table 1:** Innate immunity proteins of the Red junglefowl SF proteome.

GI #	Protein Name	# of males	Abundance percentile	Not identified in domestic chicken*
46048795	vitronectin	12	93.2	
118098426	polyubiquitin-C	11	89.8	
363744121	complement C7	12	88.0	
363740281	complement C8 gamma chain	12	86.9	
363736454	complement factor H	12	86.4	**X**
363729287	complement C4-A	12	84.0	
513213068	complement C5	12	76.9	
363740226	macrophage migration inhibitory factor	12	76.1	
221325664	complement C6	12	82.2	
513220989	complement C2 isoform X2	10	71.6	**X**
57529999	toll-interacting protein	10	60.4	
433282973	complement factor I	12	59.5	
118094713	complement C8 alpha chain	11	56.2	**X**
118094715	complement C8 beta chain	2	22.0	**X**
326633216	soluble mannose-binding lectin	1	9.6	**X**

^*^These differences may be attributable to methodological differences between this study and ref. 24 (see below).

**Table 2:** Anti-microbial and adaptive immunity proteins of the Red junglefowl SF proteome.

GI #	Protein Name	Abundance Percentile	Not identified in domestic chicken*
*Anti-microbial/viral proteins*			
48976029	gallinacin- 10	99.3	
48675889	gallinacin-9	98.8	
*Adaptive immunity proteins*			
CAA30161.1	Immunoglobulin gamma chain	99.6	
513225677	alpha-2-macroglobulin	97.0	
AAB22614.2	Immunoglobulin alpha heavy chain	96.6	
AAA48833.1	Immunoglobulin heavy chain	94.1	
P01875.2	Immunoglobulin mu chain C	91.1	**X**
513200587	beta-2-microglobulin isoform X1	82.6	
52345405	Leukocyte surface antigen CD47	71.5	
52138689	Lymphocyte antigen 86	70.5	**X**
403224973	cathelicidin-B1	68.8	**X**
448261627	ATP synthase subunit beta, mitochondrial	55.3	
113206156	N-acetylmuramoyl-L-alanine amidase	50.1	
513211517	class II histocompatibility antigen, B-L beta chain	46.5	**X**
48675879	cathelicidin-1	26.6	**X**

^*^These differences may be attributable to methodological differences between this study and ref. 24 (see below).

**Table 3:** SFPs differentially expressed with respect to male age, sperm velocity or age: velocity interaction.

Category	GI#	Protein name	Comparison or Age-velocity category
*Significant in FDR-corrected 2-way ANOVAs*			
Age: Velocity	46048932	sodium-dependent phosphate transport protein 2B	OF > YF; OF > YS
Age: Velocity	71895709	glycine-tRNA ligase	OF > YF
Age: Velocity	45382907	receptor-type tyrosine-protein phosphatase eta precursor	OF > YF
Age: Velocity	513204938	fibulin-2	OF > OS; YS > OS
Age: Velocity	160333842	proto-oncogene tyrosine-protein kinase ROS precursor	OF > OS; OF > YF; OF > YS
Age: Velocity	45382785	beta-galactoside-binding lectin	YF > YS
Age	45383337	SPARC precursor	Y > O; YF > OS; YS > OS; YS > OF
Age	303227902	acetyl-CoA acetyltransferase, cytosolic	Y > O
Age	60302752	ras-related protein Rab-11B	Y > O; YF > OS
*Identification restricted by age and/or velocity*			
Age	56605916	protein tyrosine phosphatase type IVA 1	Old only
Age: Velocity	513237884	neuroblast differentiation-associated protein AHNAK-like	Old Fast only
Age: Velocity	45383816	protein S100-A6	Old Fast only
Age: Velocity	45384028	protein S100-A11	Old Fast only
Age: Velocity	118085711	acyl-CoA-binding protein	Old Fast only
Age: Velocity	160358333	neo-calmodulin	Old Fast only
Age: Velocity	326368309	guanine nucleotide-binding protein (gamma-10)	Old Fast only
Age: Velocity	363730875	myosin regulatory light chain 2,	Old Fast only
Age: Velocity	363745926	guanine nucleotide-binding protein subunit gamma-7-like	Old Fast only
Age: Velocity	478247057	microsomal glutathione S-transferase 3	Young Fast only
Age: Velocity	363742947	nitrilase homolog 1 isoformX1	Young Fast only
Velocity	71895387	inosine-5′-monophosphate dehydrogenase 2	Slow only

OF = Old Fast; YS = Young Slow; YF = Young Fast; O = Old; S = Slow.
