# Supplementary material for: The Seminal fluid proteome of the polyandrous Red junglefowl offers insights into the molecular basis of fertility, reproductive ageing and domestication
Source: Sci Rep. 2016 Nov 2;6:35864. doi: 10.1038/srep35864 (PMC5090203; doi:10.1038/srep35864)
Supplement: Supplementary Figure S3 [file srep35864-s3.pdf]

Seminal fluid proteome of the polyandrous Red junglefowl offers insights on the molecular basis of fertility, reproductive ageing and domestication

Kirill Borziak[1*], Aitor Alvarez-Fernandez[2*], Tim Karr[3], Tommaso Pizzari[2], Steve Dorus[1†]

[1] Center for Reproductive Evolution, Department of Biology, Syracuse University, US.

[2] Edward Grey Institute, Department of Zoology, University of Oxford, UK.

[3] Drosophila Genetic Resource Center, Kyoto Institute of Technology, Saga Ippongi-cho, Ukyo-ku, Kyoto 616-8354, Japan.

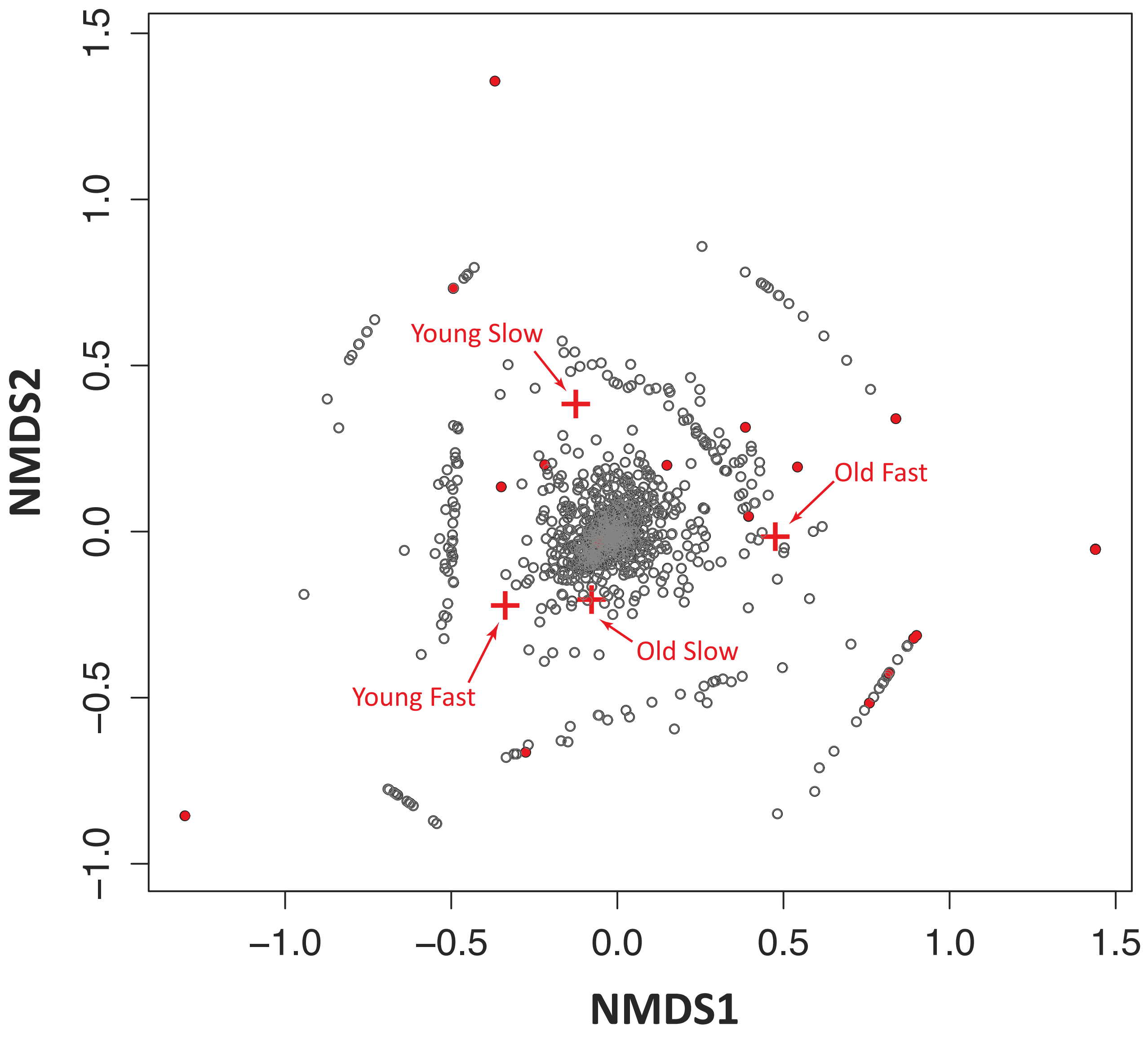

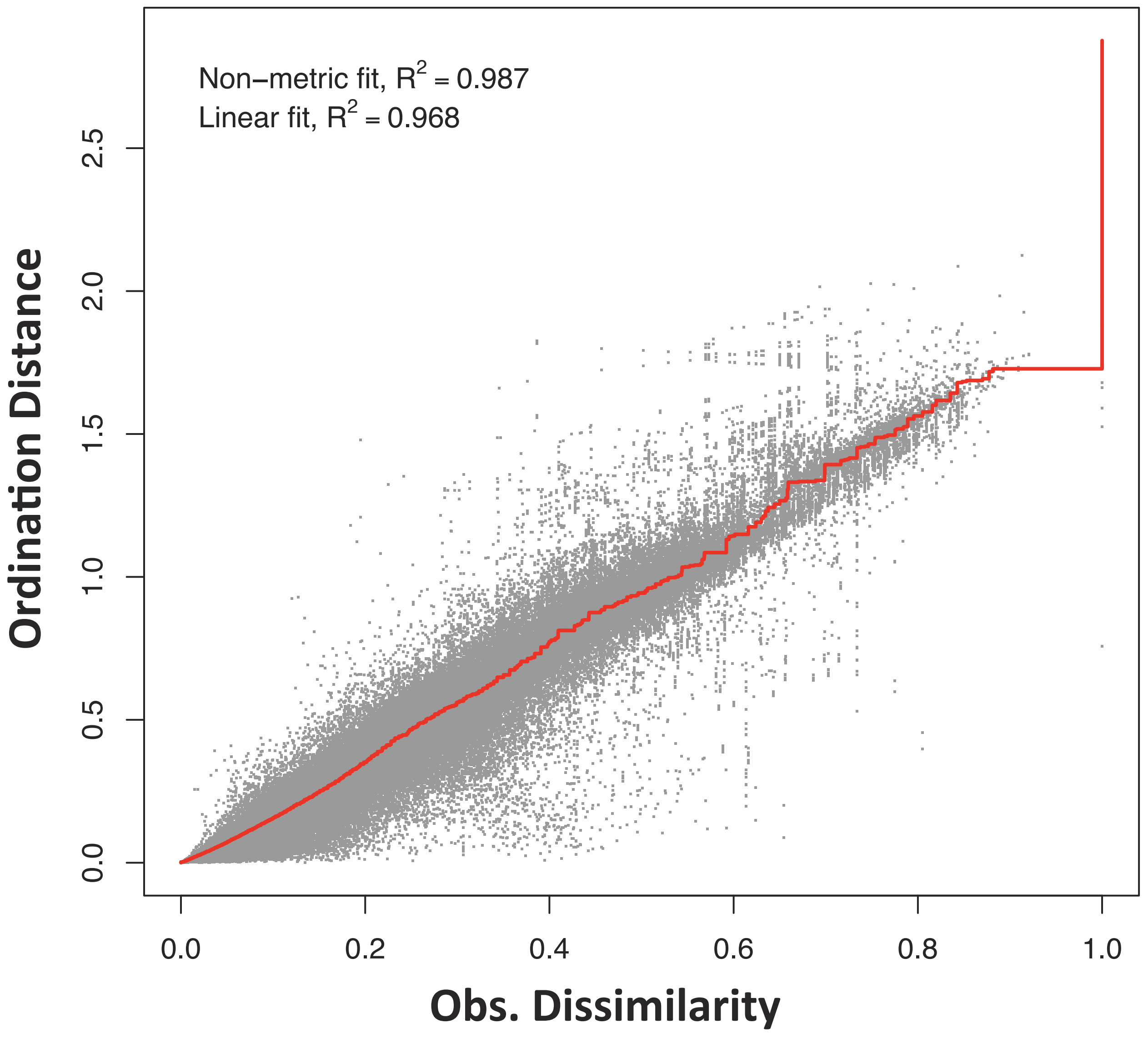

Supporting Material Figure S3. (A) Non-metric multidimensional scaling (NMDS) biplot representing the four categories in a 2-dimensional ordination space. Proteins with a significant age:velocity effect in the two-way ANOVA are indicated in red; the weighting of each male cohort is indicated by a "+". (B) Shepard's Stress Plot indicating the relationship between the original protein distances and their dissimilarities in the final two-dimensional ordination space.
