# Supplementary material for: The Seminal fluid proteome of the polyandrous Red junglefowl offers insights into the molecular basis of fertility, reproductive ageing and domestication
Source: Sci Rep. 2016 Nov 2;6:35864. doi: 10.1038/srep35864 (PMC5090203; doi:10.1038/srep35864)
Supplement: Supplementary Figure S5 [file srep35864-s5.doc]

**Supplemental Material S5**. (**A**) Frequency distribution of Average Path Sperm Velocity (VAP) in the male junglefowl population across different ages: 7 young (1 yr old), 7 intermediate age (3 yr old), and 9 old (4, 5 and 7 yr old) males. Each male was sampled between 4 and 13 times throughout the reproductive season by abdominal massage, for a total of 228 samples. (**B**) Average Path Sperm Velocity (VAP) of the samples selected for proteomic analysis across the four categories, Young Fast, Young Slow, Old Fast and Old Slow. Means and standard errors (vertical bars) for each category were calculated using the average of the 3 samples from each of the three males in the category. There was a significant difference between Young and Old males (2-way ANOVA on Log_10_(VAP), Age: *F*_1, 32_=12.906, *p*= 0.0011) and between Fast and Slow samples (Velocity: *F*_1, 32_=293.47, *p*<2.2e-16), but no Age:Velocity interaction (*F*_1, 32_=0.166, p=0.686).

**Title: Seminal fluid proteome of the polyandrous Red junglefowl offers insights on the molecular basis of fertility, reproductive ageing and domestication**

Kirill Borziak^1*^, Aitor Alvarez-Fernandez^2*^, Tim Karr^3^, Tommaso Pizzari^2^, Steve Dorus^1†^

^1^ Center for Reproductive Evolution, Department of Biology, Syracuse University, US.

^2^ Edward Grey Institute, Department of Zoology, University of Oxford, UK.

^3^ Drosophila Genetic Resource Center, Kyoto Institute of Technology, Saga Ippongi-cho, Ukyo-ku, Kyoto 616-8354, Japan.
